# Virtual Cold Chain Method to Evaluate the Effect of Rising Temperature on the Quality Evolution of Peach Fruit

**DOI:** 10.3390/foods12122403

**Published:** 2023-06-17

**Authors:** Hui Liu, Zhenzhen Lv, Wenbo Yang, Ang Li, Jiechao Liu, Qiang Zhang, Zhonggao Jiao

**Affiliations:** 1 Zhengzhou Fruit Research Institute, Chinese Academy of Agricultural Sciences, Zhengzhou 450009, China; liuhui@caas.cn (H.L.); lvzhenzhen@caas.cn (Z.L.); yangwenbo@caas.cn (W.Y.); 1125351664la@gmail.com (A.L.); liujiechao@caas.cn (J.L.); jiaozhonggao@caas.cn (Z.J.); 2 Zhongyuan Research Center, Chinese Academy of Agricultural Sciences, Xinxiang 453000, China

**Keywords:** virtual cold chain, temperature abuse, peach fruit, fruit core temperature, fruit quality

## Abstract

Poor temperature management along a cold chain leads to fruit quality deterioration and loss. In order to determine the threshold value of temperature fluctuation in a cold chain, peach fruits were stored in four different virtual cold chains applying different temperature–time scenarios. Core temperature profiling, the physicochemical qualities, and the activities of the peaches’ antioxidant enzymes were monitored during cold storage and shelf life. Abusive temperature management (temperature increased to 20 and 15 °C three times) resulted in a significant increase in a peach’s core temperature to the highest temperature measured: 17.6 °C. The ethylene production rate at the end of the shelf life of peaches under these temperatures was 21.03–28.16% higher than the constant-temperature group and accompanied by significantly lower levels of flesh firmness, titratable acid content, total phenol and flavonoid content, and peroxidase (POD) and catalase (CAT) activities (*p* < 0.05). The results of a principal component analysis (PCA) and heatmap confirmed the results. Limited temperature increases (10 °C) in a cold chain had little impact on the quality of the peaches, while temperature increases higher than 15 °C three times would negatively affect the quality of the peaches significantly. The temperature of a cold chain needs to be controlled precisely to reduce the loss of peaches.

## 1. Introduction

The production, consumption, and exporting of fruits constitute a major sector of the Chinese economy. The daily average consumption of Chinese residents is in the tens of thousands of tons; however, production is not fully utilized as there exist tremendous losses and waste in the postharvest stage [1]. Due to the characteristics of fragile and perishable fruits—which are greatly affected by temperature, regional production, and the timeliness of consumption—they are prone to a decline in quality during the postharvest circulation process, causing great economic losses. According to an estimate of food loss in China, 10–15% of the loss of fruits occurs at the storage stage, while 5–10% occurs at the distribution stage [2].

An effective way to solve this problem is cold chain circulation, a logistics network equipped with special equipment to maintain the quality of fresh food in a low-temperature state from producer to consumer; however, ambient-temperature transportation and incomplete cold chain circulation are still commonly used for fruit and vegetable supplies in China. Almost 95% of vegetables and fruits are transported via trucks without refrigeration [3]. The amount of postharvest decay of fruits and vegetables caused by non-cold chain circulation is huge. It is very important to clarify the impact of incomplete cold chain supply on the quality of fruits and vegetables.

Temperature is the most important environmental parameter in a cold chain, and it has received much attention because unexpected temperatures can lead to food safety issues and the loss of quality [4,5,6,7]. Much research has focused on the impact of non-optimal temperatures in some stages of the food supply chain on the quality of fruits, including the precooling, handling operation, distribution store, and consumer refrigeration stages [8,9,10,11]. Kelly et al. [12] and Ktenioudaki et al. [13] reported that the different temperatures used in the critical steps of a supply chain had different effects on the quality of strawberries and blueberries. Their results showed that the maintenance of a constant, optimum temperature throughout a simulated supply chain is paramount for limiting the loss of fruit quality. Temperature fluctuations during storage or transportation also influenced fruit quality according to some research. Delayed precooling, long transit times, and fluctuating temperatures encountered during the handling of strawberries from the field to the store contributed to poor fruit quality [14]. Temperature fluctuations also accelerated the color change and softening of postharvest sweet cherries [7]. Redier et al. [15] reported that large temperature fluctuations significantly increased the total number of *Escherichia coli* and *Enterobacteriaceae* bacteria in the fresh-cut endive.

Nowadays, the temperature of each facility can be easily controlled due to the progress in refrigeration technology; however, some steps are especially hard to control—including handling, transportation, loading, and unloading. Difficulty maintaining a constant, optimum temperature all along a cold chain is a major cause of fruit and vegetable deterioration in appearance and nutritional value [5,12,16]. To maintain fruit quality and reduce these losses, maintaining an optimum cold temperature throughout a supply chain is very important. Once the fruits are picked up from their place of origin, they pass through distributors, processing plants, wholesalers, and urban fruit cold storages in stages, and then they finally reach consumers [17] (pp. 1–4). During these stages, the transfer of the fruits between different facilities results in temperature changes due to their handling and environmental changes, which may even cause the chain to break.

Any break in a cold chain will influence food quality or cause a loss of food quality [1]. Effective cold chain temperature management is difficult to achieve, and fresh produce is regularly subjected to abuse temperatures of 8–12 °C [15]. One reason for this is that produce is usually exposed to an elevated temperature when it is transferred from one operation unit to another [4]. When temperatures rise, the fruit and vegetable often have to be re-cooled several times throughout a cold chain journey. Nowadays, few studies have focused on the effects of different environmental conditions on the quality of fruits when they were transferred between different facilities. In our previous research, we monitored peach core temperature profiling when the transfer stage temperature rose to 25 °C, and it was confirmed that the increase in temperature negatively affected peach fruit quality compared to the constant-temperature condition at 4 °C, including the firmness, content of soluble sugars, organic acids, and cell wall polysaccharides [18]. There is an urgent need to evaluate the effects of different transfer temperatures on fruit quality. The results will help to improve the accurate parameters along fruit cold chains.

Although some studies have shown that fruit quality was significantly affected by the abuse of temperature in different facilities in the supply chain, there is no information available about the impact of rising temperature in the transfer stages on fruit quality and about the threshold value of temperature fluctuations. The objective of this research was to evaluate the effects of temperature abuse in transfer stages between different facilities on fruit quality and to confirm the temperature-controlled threshold value. Comparable postharvest treatments and transporting conditions are often difficult to replicate when conducting real-life experiments. Repetitions are not always possible [14] and virtual experiments need to be executed. Peach fruit was chosen as the research material and was stored in a simulated cold chain under different temperature conditions. The fruit core temperature, ethylene production rate, respiratory rate, flesh firmness, MDA, SSC, TA, total polyphenol and flavonoid content, and antioxidant enzyme activity throughout different virtual supply chains were measured. Additionally, the temperature increase threshold value during the transfer stage in a cold chain that affected fruit quality was determined. The results could help to improve adequate cold chain parameters and preserve fruit with high quality.

## 2. Materials and Methods

### 2.1. Description of Virtual Supply Chains

**Figure 1:**
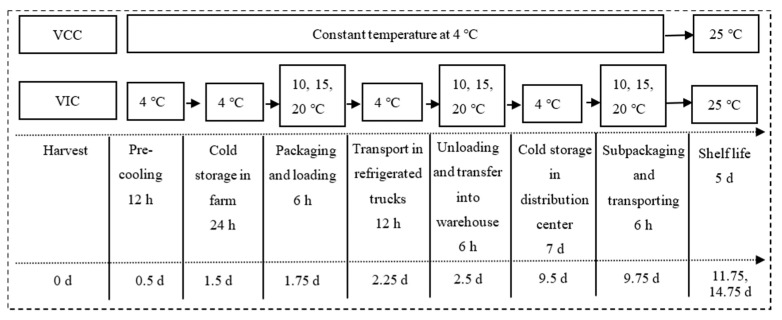
The temperature changes in the virtual complete cold chain (VCC) and virtual incomplete cold chain (VIC) of peach fruit. d, day.

The supply chain was divided into nine stages from the producer to the consumer, as shown in Figure 1. After harvesting, the fruits were precooled at 4 °C for 12 h and stored in the orchard for 1 day to simulate a situation where the fruits are unsold on the same day. The fruits were then graded, packaged, and transferred out of cold storage; this was the first instance of temperature deviation involving the handling operation (1.75 day). After the 12 h of transportation in a refrigerated vehicle, the fruits were unloaded and transferred into a cold warehouse in the distribution center (DC). This process saw the second instance of an increase in temperature (2.5 day). Finally, after being stored for 7 days in the DC cold warehouse, the fruits were subpackaged and transported to the terminal sale store, where the temperature increased for the third time (9.75 day). The retail store’s and consumer’s storage temperatures were set to 25 °C for 5 days to simulate shelf life.

There are four treatments simulating supply chains with different temperature conditions. One is a virtual complete cold chain (VCC); the temperature along the whole cold chain was at an isothermal 4 °C. For the virtual incomplete cold chain (VIC) groups, the peaches were transferred to 10, 15, and 20 °C for 6 h in three transfer stages to simulate the handling, loading, and unloading processes along the supply chain, noted as the VIC-10, VIC-15, and VIC-20 groups, respectively. The temperature of other stages of the VIC was consistent with the VCC group at 4 °C.

### 2.2. Material and Sample Preparation

The “Chunmei” peach fruit was harvested at commercial maturity from an orchard in Yuanyang County of Henan Province in June 2022, and transported to the laboratory within 3 h after harvest. Fruits with the same size, no disease and insect pests, and no mechanical damage were selected as the materials.

A total of 660 fruits were randomly divided into 4 groups with 3 replicates in each group. Every 5 fruits were packaged in a polyethylene plastic bag with a thickness of 0.22 mm. The relative air humidity was 90~95% in the cold warehouse. For each replicate, 5 fruits were used to monitor the fruits’ core temperature during the cold chains. At each sampling time, 5 fruits of each replication were randomly sampled and used to measure the respiration rate, ethylene production rate, and flesh firmness. The fruit flesh was then cut into small slices, frozen in liquid N_2_, and stored at −80 °C for the measurement of SSC, TA, MDA, total phenol and flavonoid content, and enzyme activity.

The ethylene production rate and respiratory rate of peaches were measured at all of the stages in Figure 1. The critical stages of the virtual cold chains involving temperature changes were the sampling points for fruit quality analyses, including at harvesting (0 day), after packaging and loading from the farm (1.75 day), after transporting and unloading into the DC warehouse (2.5 day), after transporting from the DC to the terminal sales (9.75 day), and day 2 and day 5 of shelf life (11.75 and 14.75 day).

### 2.3. Fruit Core Temperature

The fruit core temperature was measured by time–temperature data loggers (L95-2, HangZhou Loggertech Co., Ltd., Hangzhou, China). The probe of the data loggers was inserted 1 cm deep into the flesh of peach fruit and the value was recorded every 1 h.

### 2.4. Respiratory Rate and Ethylene Production Rate

Peach fruits were put at 25 °C for 2 h before the test to achieve fruit temperature equilibrium [19] to eliminate the impact of environmental temperature on the results. The fruits were then sealed in a container connected to a fruit and vegetable respiration tester (SY-1022, Shijiazhuang Shiya Technology Co., Shijiazhuang, China) at 25 °C for 1 h, and the result was expressed as mg CO_2_/kg·h. Ethylene production was measured by a gas chromatograph (GC 2010, Shimadzu Co., Ltd., Kyoto, Japan). The fruits from each replicate were put into an airtight chamber for 2 h, after which a 0.5 mL gas sample was injected into the GC equipped with a flame ionization detector (FID) and an alumina Al_2_O_3_/S column (30 m × 0.53 mm, 0.25 μm, Welch Tech. Co., Ltd., Shanghai, China). The ethylene concentration (μL/L) was quantified using the standard curve. Additionally, the ethylene production rate was expressed as μL/kg·h.

### 2.5. Firmness, SSC and TA Content 

Fruit firmness was measured on the two vertical sides of the fruit equator without peel via using a texture tester (TA-XT plus, Stable Micro Systems Ltd., Godalming, UK) fitted with a probe with a diameter of 5 mm. The test speed was 1 mm/s, and the penetration depth was 5 mm. The data were expressed as kg/cm^2^. The fruit flesh was juiced to test the soluble solid content (SSC) with a handheld saccharometer (PAL-1, ATAGO Co., Ltd., Tokyo, Japan). TA content was measured via titrating with 0.05 mol/L NaOH.

### 2.6. Malondialdehyde (MDA) Content

MDA content was measured according to Dhindsa et al. [20], with some modifications. One gram of fruit flesh was ground in 3 mL of 10%(*w*/*v*) trichloroacetic acid (TCA). After centrifugation at 12,000× *g* for 15 min at 4 °C, 1 mL of supernatant was mixed with 1 mL of 0.67% (*w*/*v*) thiobarbituric acid (TBC) and 2 mL of TCA. The mixture was incubated in boiling water for 20 min. After cooling to the room temperature, the absorbances of 450, 532, and 600 nm were measured via a spectrophotometer (Specord 50, Analytik Jena Instrument Co., Ltd., Jena, Germany). The MDA content was calculated according to the following formula: MDA (μmol/g) = [6.45 × (A_532_ − A_600_) − 0.56 × A_450_] × V/m.

### 2.7. Total Phenol and Total Flavonoid Content

The homogeneous flesh tissue (1 g) was mixed with 10 mL of methanol and extracted for 10 min via an ultrasonic bath at 30 °C. After being centrifuged at 12,000× *g* at 4 °C for 15 min, the supernatant was collected and used to determine the total phenol content (TPC) and total flavonoid content (TFC). For the measurement of TPC, 0.5 mL of supernatant was added into 2.5 mL of Folin–Ciocalteu reagent (prediluted 50-fold) and the mixture was incubated at 50 °C for 5 min. After the test tube was cooled to room temperature, 2 mL of sodium carbonate (7.5%, *w*/*v*) was added and left for 30 min. The absorbance of 760 nm was then measured [21]. Gallic acid was used as the standard, and the TPC result was expressed as mg GA/100 g. To measure the TFC, 1.0 mL of supernatant was mixed with 0.3 mL of sodium nitrite (5.0 %, *w*/*v*) and left for 6 min, after which 0.3 mL of 10% (*w*/*v*) aluminum nitrate was added and left for 6 min, followed by 2.4 mL of 1.0 mol/L sodium hydroxide. The absorbance of 510 nm was measured after 15 min. Catechin was used as the standard and the TFC was expressed as mg CA/100 g.

### 2.8. SOD, POD, and CAT Activity

Catalase (CAT), superoxide dismutase (SOD), and peroxidase (POD) activity was measured using assay kits (Beijing Solarbio Technology Co., Ltd., Beijing, China) according to the directions of the manufacturers. Enzyme activity was expressed as U/g.

### 2.9. Statistical Analysis

The experiments were laid out in a completely randomized design and the results were shown as means ± standard deviations (SDs). An analysis of variance (ANOVA) was performed and the means were compared with Tukey tests through using OriginPro 2021 (OriginLab Co., Northampton, MA, USA). A principal component analysis (PCA) and biplot were also performed through OriginPro 2021. A heatmap analysis was created with the website of ClustVis 2.0 [22].

## 3. Results and Discussion

### 3.1. Fruit Core Temperature Changes in Different Virtual Cold Chains

**Figure 2:**
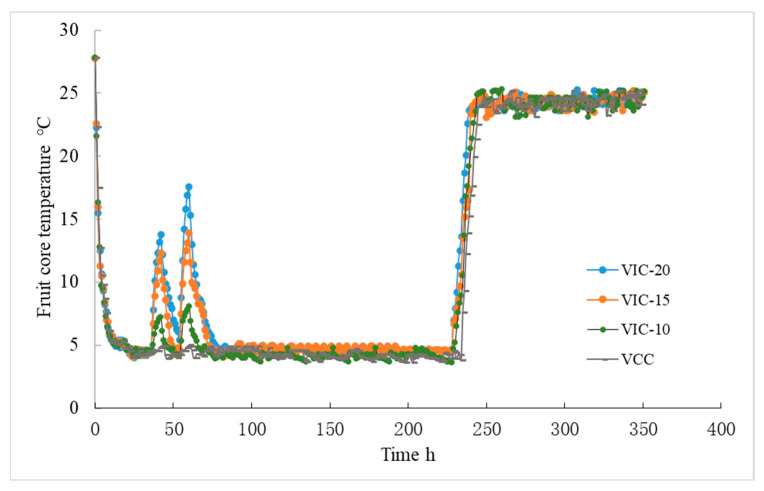
Peach fruit core temperature changes in different virtual cold chains. The virtual incomplete cold chains with ambient temperatures rising to 20, 15, and 10 °C are noted as VIC-20, VIC-15, and VIC-10, respectively. VCC means a virtual complete cold chain with a constant temperature.

Monitoring the postharvest fruit temperature history is important for evaluating the efficacy of existing cold chain unit operations but also for exploring new cooling strategies [5]. Figure 2 showed the changes in the fruit core temperature along the different simulated cold chains. The temperature decreased to 5.1–5.4 °C after 12 h of precooling. When the fruits were graded, packaged, and transferred outside the farm cold warehouse (1.5–1.75 d), the fruit core temperature increased to 13.8, 12.3, and 7.2 °C as the ambient temperature rose to 20, 15, and 10 °C, respectively. The peaches were then cooled again to simulate transportation in a refrigerated truck. After this, the temperature was then raised for a second time to simulate the fruit being transferred from the truck into a DC cold warehouse (2.25–2.5 d). The fruit core temperature increased to 17.6, 13.9, and 8.1 °C, respectively, in the VIC-20, VIC-15, and VIC-10 groups. The fruit core temperature (17.6 °C) of the VIC-20 group after the second instance of an increase in temperature was higher than that of the first time (13.8 °C). This may be due to the fact that the initial fruit core temperature (5.4 °C) was higher, which was due to the fact that the re-cooling time was not enough for adequate cooling; the cooling of fruit by the cold warehouse demanded a longer time [1]. Meantime, the outside temperature would affect the cooling time, as reported by Rediers et al. [15]. To reach the same cooling temperature (10 °C), the time used for a warm outdoor temperature (14–35 °C) was 1.5-fold that at a moderate temperature (5–19 °C) [15]. After being stored in the DC warehouse for 7 days, the peach fruits were subpackaged and transported to the shelf for sale, where the fruits experienced the third instance of an increase in temperature (9.5–9.75 d). The fruit core temperature increased to 13.6, 10.6, and 8.9 °C, respectively, in the VIC-20, VIC-15, and VIC-10 groups. The effect of different ambient temperature conditions on the fruit core temperature fluctuation was obvious. The higher the ambient temperature increased, the faster the fruit core temperature rose and the longer the times needed for fruit re-cooling. The fruit core temperature of peaches stored in the virtual cold chain with temperature rising to 10 °C (VIC-10) increased slightly (7.2–8.9 °C).

### 3.2. Respiratory Rate and Ethylene Production Rate of Peach Fruit in Different Virtual Cold Chains

**Figure 3:**
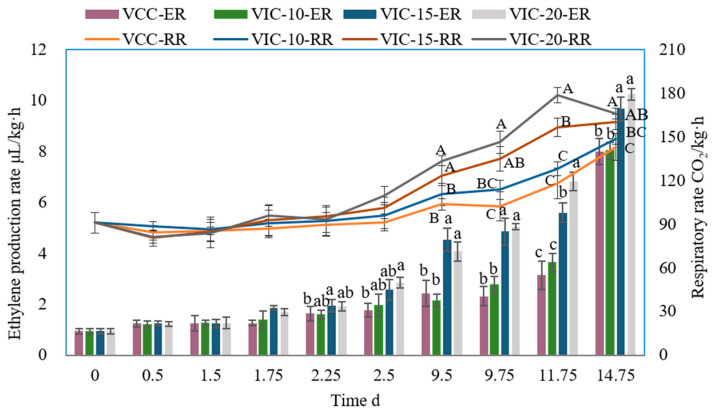
The ethylene production rate (ER) and respiration rate (RR) of peach fruit stored in different virtual cold chains. The virtual incomplete cold chains with ambient temperatures rising to 20, 15, and 10 °C are noted as VIC-20, VIC-15, and VIC-10, respectively. VCC means a virtual complete cold chain with a constant temperature. Different uppercase and lowercase letters mean significant differences in the respiratory rate and ethylene production rate among different groups (*p* < 0.05), respectively.

Respiration rate and ethylene production rate are closely related to fruit maturity after harvesting [23]. The changes in the peach fruit ethylene release rate and respiration rate along the virtual cold chain were analyzed, and the results were shown in Figure 3. As shown in Figure 3, the ethylene production rate and respiration rate increased slowly during the cold storage stage (0–9.75 day), but they increased rapidly when the fruits were transferred to shelf life (11.75–14.75 day). The results were similar to those of other studies on peaches [24]. The ethylene release rate and respiration rate were significantly affected by the ambient temperature and represented a regulatory effect on postharvest peach fruit quality [25].

After the first instance of an increase in the ambient temperature (1.75 d) to 15 °C, the ethylene release rate (1.85 µL/kg·h) was significantly higher than that of the constant-temperature group (1.26 µL/kg·h). After the second instance of an increase in temperature (2.5 d), the ethylene production rate significantly increased to 2.84 µL/kg·h of the VIC-20 group compared to 1.76 µL/kg·h of the constant-temperature group. The significant difference in the ethylene release rate between the VCC, VIC-20, and VIC-15 groups still existed from 9.5 d to the end of shelf life. No significant differences in the ethylene production rate appeared between the VIC-10 group and constant-temperature group during the virtual cold chain. Other research has reported that heat treatment significantly increased ethylene production during peach storage because of the availability of high levels of ACC (the precursor of ethylene synthesis) [26].

The respiratory rate showed a similar trend as the ethylene production rate, but not exactly the same. The respiratory rate showed no significant difference among the different virtual cold chains before 9.5 d; however, after the third instance of an increase in temperature (9.75 d), the respiratory rate of VIC-20 and VIC-15 increased to 134.97 mg CO_2_/kg·h and 146.31 mg CO_2_/kg·h, which were significantly higher than that of the constant-temperature group. At the end of shelf life, the peach respiratory rate of the VIC-15 and VIC-20 groups was 12.36% and 15.89%, respectively, higher than that of the VCC group. The results show that ambient temperature, which increased to 15 °C and 20 °C three times during the supply chain, could significantly promote the respiratory metabolism of peach fruit. Higher temperatures always resulted in faster respiratory rates of peaches [27].

### 3.3. Firmness of Peach Fruit in Different Virtual Cold Chains

**Figure 4:**
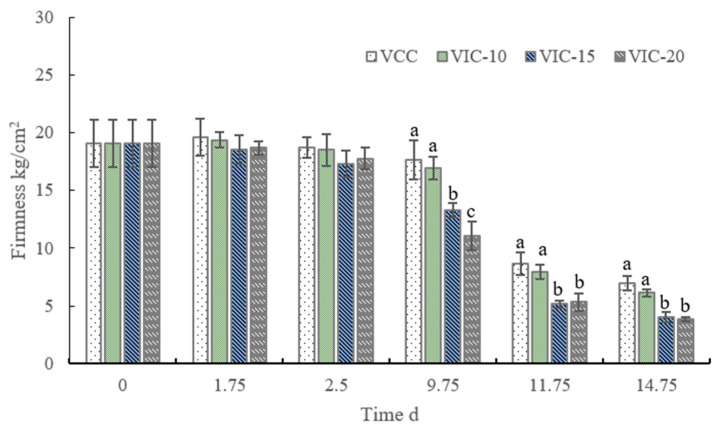
Firmness changes in peach fruit stored in different simulated cold chains. The virtual incomplete cold chains with ambient temperature rising to 20, 15, and 10 °C are noted as VIC-20, VIC-15, and VIC-10, respectively. VCC means a virtual complete cold chain with a constant temperature. Different letters mean significant differences among different groups (*p* < 0.05).

Firmness is an important quality attribute that affects consumer acceptance, and flesh softening is one of the main performance indicators of a decline in storage properties. As shown in Figure 4, the flesh firmness of peach fruit gradually decreased during the cold storage stage (0–9.75 d). There was no significant difference among the different virtual cold chains from 0 d to 2.5 d; however, after the peaches were stored for 7 days in the DC and then experienced the third instance of an increase in temperature, the fruit firmness of the VIC-10 (16.95 kg/cm^2^) and constant-temperature groups (17.62 kg/cm^2^) were significantly higher than the VIC-15 and VIC-20 groups. The peach fruit softened quickly when in shelf life; similar results were reported by Cai et al. [24] for peaches. At the end of shelf life, peaches of the constant-temperature and VIC-10 groups also showed significantly higher firmness.

Previous studies have shown that the softening of peaches is temperature-dependent. Fruit firmness significantly decreased when the fruit was stored at higher temperature of 8–15 °C compared to lower temperature [25]. The temperature fluctuation during the strawberry supply chain resulted in lower firmness compared to the semiconstant-temperature condition [8,9]. Nunes and Emond [8] found that, after 6 d of storage, strawberries at a constant temperature were 6% less firm than the initial samples, while those fruit stored under a fluctuating temperature were about 11% less firm. Similar results were reported by Kelly et al. [12], insofar that strawberries at an optimum constant temperature along the whole supply chain were firmer than fruit from other (higher temperature or delayed cooling) scenarios.

High temperatures induced fruit softening, commonly accompanied by an increase in cell wall hydrolysis enzyme activity and the modification of the cell wall composition, which were impacted by the release of ethylene and respiration [7,25]. The increase in ambient temperature to 15 °C and 20 °C promoted the production of ethylene and respiration, as shown in Figure 3. Respiration can result in chemical and enzymatic changes and cause tissue softening, ripening, or discoloration [15]. Ethylene can regulate softening-related enzymes and result in the ripening of peach fruit [28,29]. The rising of fruit core temperature could also activate cell-wall-degrading enzymes and promote the degradation of cell wall polysaccharides. Low temperatures could decrease enzyme activity, delaying the softening of peach fruit [30]. Some researchers reported that a halfway temperature increase or temperature fluctuation during storage could increase the activity of PME and PG, accelerating the degradation of pectin and fruit softening [7,31]. In our previous research [18], when the ambient temperature rose to 25 °C during the supply chain the peach cell wall pectin content changed significantly. In particular, sodium-carbonate-soluble pectin decreased and water-soluble pectin increased significantly at the end of shelf life. The changes in cell wall degradation enzyme activity and cell wall polysaccharide structure under different conditions concerning rising temperature should be studied in other research to reveal the mechanism of its regulation of fruit firmness.

### 3.4. SSC and TA Content of Peach Fruit in Different Virtual Cold Chains

**Figure 5:**
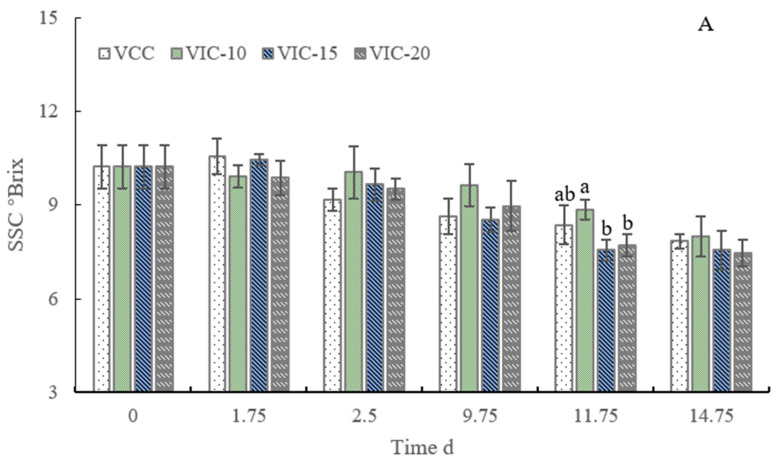

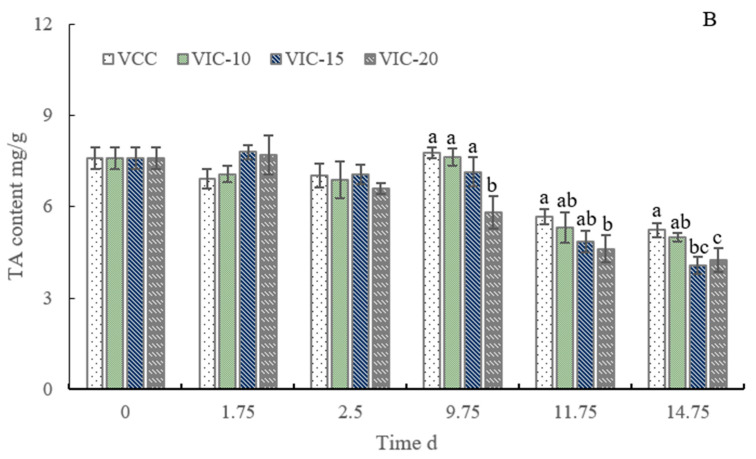
SSC (**A**) and TA content (**B**) of peach fruit stored in different virtual cold chains. The virtual incomplete cold chains with ambient temperature rising to 20, 15, and 10 °C are noted as VIC-20, VIC-15, and VIC-10, respectively. VCC means a virtual complete cold chain with a constant temperature. Different letters mean significant differences among different groups (*p* < 0.05).

As shown in Figure 5A, there were no significant differences in SSC among different virtual cold chain groups, except for the SSC of the VIC-10 group, which was higher than that of the VIC-20 and VIC-15 groups on day 2 of shelf life. The higher respiratory rate of the VIC-20 and VIC-15 groups would consume more sugars as the substrate. Furthermore, the temperature could affect the composition and content of sugars in the fruits by regulating the activity of enzymes related to sugar metabolism. Wang et al. [32] found that the activity of neutral invertase (NI) and acid invertase (AI) of peaches stored at 5 °C was higher, but that of sucrose phosphate synthase (SPS/1) was lower, compared to 0 °C storage, and the higher temperature accelerated the degradation of sucrose. Our previous research on peaches stored in different simulated supply chains [18] found that the contents of reducing sugar and soluble sugar were significantly affected by the different temperature conditions. When the transfer temperature increased to 25 °C in the simulated incomplete cold chain, it promoted a decrease in sucrose content and SSC. Nunes et al. [8] found that the glucose content was lower in the fruits from the fluctuating temperature treatment than those under a constant temperature. In other research, the SSC of strawberries shipped under the fluctuating temperature was lower than that of the constant temperature [14]. Additionally, a decrease in fruit SSC had been reported when the fruit was handled under a higher temperature due to the depletion of the sugar reserves that resulted from an increase in fruit respiration metabolism involving the consumption of simple sugars [33].

The TA content of peaches decreased in shelf life, similarly to what the report of Cai et al. [24] found for peach fruit. The TA content of the VIC-20 group decreased quickly after 7 days of storage in the distribution center, which was significantly lower than that of the constant-temperature group from 9.75 d to 14.75 d. A significant difference was also shown between the VIC-15 group and the constant-temperature group on day 5 of shelf life. The increase in temperature during the transfer stages significantly impacted the organic acid metabolism. The acidity of strawberries was less reserved at higher temperatures during the supply chains compared to the constant-temperature condition and the initial fresh fruits [12]. Nunes et al. [8,9] also reported that, compared to the control constant-temperature condition, the strawberries exposed to the simulated supply chain under temperature-fluctuation conditions possessed lower contents of acidity. Our previous research also analyzed the change in organic acid content in different simulated supply chains. The results showed that malic acid and citric acid were the major organic acids in peaches and that they decreased along the supply chain [18]. The content of malic acid was significantly lower in the incomplete cold chain group than the constant-temperature group in shelf life [18]. Other researchers also found that an increase in ambient temperature could result in a decrease in TA and malic acid content [34,35]. An increase in temperature could also alleviate the accumulation of malic acid and decrease the production of citric acid in ripening fruit [36,37]. Maybe this was due to the improvement caused by the higher temperature in terms of enzyme activity that affected glycolysis and the TCA cyclic reaction [38].

### 3.5. MDA Content of Peach Fruit in Different Virtual Cold Chains

**Figure 6:**
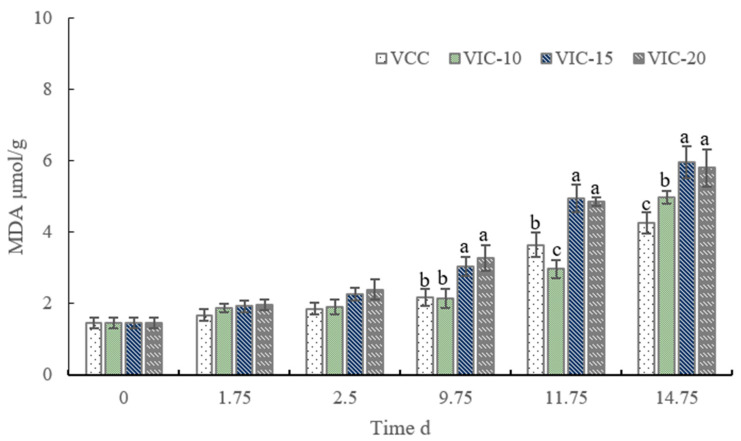
MDA content of peach fruit stored in different virtual cold chains. The virtual incomplete cold chains with ambient temperatures rising to 20, 15, and 10 °C are noted as VIC-20, VIC-15, and VIC-10, respectively. VCC means a virtual complete cold chain with a constant temperature. Different letters mean significant differences among different groups (*p* < 0.05).

MDA is an end lipid oxidant product and used as an indicator of lipid peroxidation and loss of membrane integrity. The senescence stress of fruit during storage is generally accompanied by the accumulation of MDA [39]. As shown in Figure 6, the MDA content of peach fruit in different simulated cold chains increased significantly from 1.46 µmol/g initially, at 0 d, to 4.27~5.97 µmol/g at the end of shelf life. The MDA content changed differently in the four temperature conditions, suggesting that increases in temperature to different values affected the senescence of peach fruit differently. When the temperature increased to 20 °C and 15 °C in the third instance at 9.75 d, the MDA content increased to 3.27 and 3.05 µmol/g, which was significantly higher than that of the constant-temperature and VIC-10 groups. The significant difference was sustained until the end of shelf life. An et al. [27] compared peach fruit apoptosis-related physiological factors under different storage temperatures; the MDA content of fruits stored at 25 °C was much higher than those stored at 4 °C. The accumulation of MDA in peaches of the VIC-20 and VIC-15 treatments was consistent with fruit softening, due to the more serious destruction of the cell wall and cell membrane integrity. An increase in temperature always enhanced lipid peroxidation with MDA accumulation in the vegetable and fruit during postharvest storage [40].

### 3.6. Total Phenol and Flavonoid Content of Peach Fruit in Different Virtual Cold Chains

**Figure 7:**
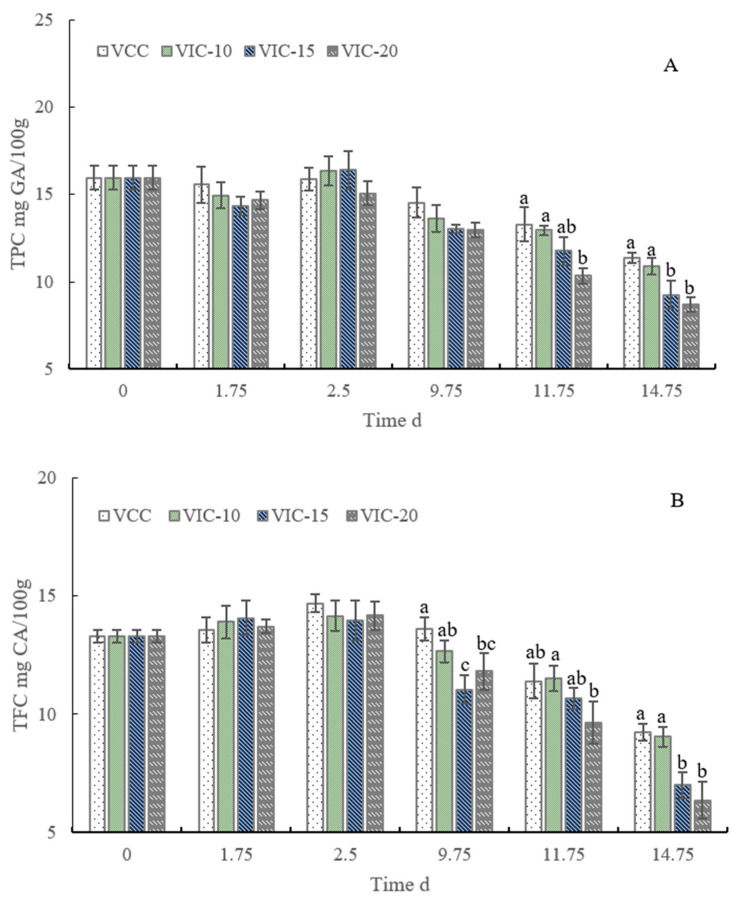
Total phenol content (**A**) and flavonoid content (**B**) of peach fruit stored in different virtual cold chains. TPC: total phenol content; TFC: total flavonoid content. The virtual incomplete cold chains with ambient temperatures rising to 20, 15, and 10 °C are noted as VIC-20, VIC-15, and VIC-10, respectively. VCC means a virtual complete cold chain with a constant temperature. Different letters mean significant differences among different groups (*p* < 0.05).

Polyphenols are directly involved in plant stress resistance and also affect the nutritional and storage properties of fruit. As shown in Figure 7, the TPC and TFC both increased slightly in the earlier stage and then decreased with the prolonging of storage time. The increase in total phenolic and flavonoid content may be caused partly by the cold temperature stress response. A decrease in total phenol content during peach storage has been reported in several studies [41,42]. The MDA content increasing remarkedly indicated the damage of the membrane integrity of fruit tissue. The collapse of cellular membranes allowed the degrading enzymes, such as polyphenol oxidase (PPO) and POD, to come into contact with the polyphenols and to catalyze the oxidation of phenolic compounds to quinines and result in a decrease in total polyphenol content [43,44].

There were some differences in the TPC and TFC changes in peaches along the different simulated cold chains. At 9.75 d of storage, the peaches stored under a constant temperature possessed more flavonoids (13.59 mg/100 g) than the VIC-15 and VIC-20 groups, while the TPCs showed no significant differences. At day 2 of shelf life, the TPC of the VIC-20 group was significantly lower than that of the VCC group, but the difference of TFC did not exist. At the end of shelf life, both the TPC and TFC of the VIC-20 and VIC-15 groups were significantly lower than that of the VIC-10 and VCC groups. The increase in transfer temperature to higher than 15 °C three times accelerated the loss of polyphenols in peaches. Higher temperatures usually reduced fruit phenols as storage time continued. Kelly et al. [12] compared the different supply chain steps with different temperatures and found that strawberries shipped to stores at 8 °C showed the largest decrease in total phenolics; additionally, fruits being stored in the distribution center at 5 °C also impacted the total phenolics. Increases in the ambient temperature resulted in the destruction of the cell membrane and accelerated the reaction process of polyphenols with oxidase, then reducing the content of polyphenols. Some researchers reported that ascorbic acid content decreased in strawberries exposed to the simulated different temperature conditions compared to the constant temperature [9,12]; however, the results showed that the total phenolic contents under some temperature abuse conditions were higher than those of the constant-temperature group, which was possibly caused by the stress response to adverse handling conditions [12]. The different results of our research might be due to the different fruit materials and different simulated temperature–time conditions. The increment of the total phenolic content induced by the stress response in peaches was much lower than its oxidation and reduction during storage and shelf life.

### 3.7. SOD, POD, and CAT Activities of Peach Fruit in Different Virtual Cold Chains

**Figure 8:**
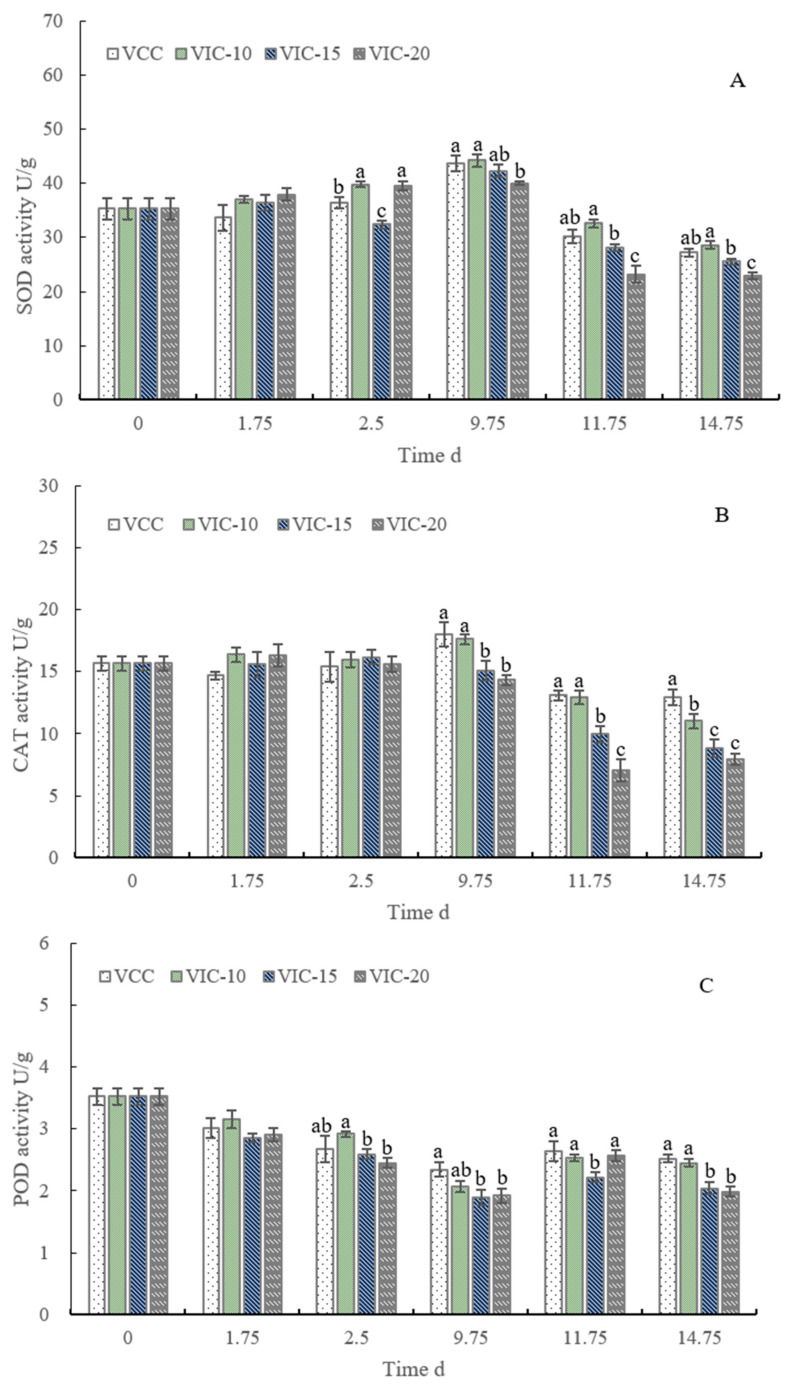
Activities of SOD (**A**), CAT (**B**), and POD (**C**) of peach fruit stored in different virtual cold chains. The virtual incomplete cold chains with ambient temperatures rising to 20, 15, and 10 °C are noted as VIC-20, VIC-15, and VIC-10, respectively. VCC means a virtual complete cold chain with a constant temperature. Different letters mean significant differences among different groups (*p* < 0.05).

SOD, POD, and CAT form a complete antioxidation chain and play important roles in preventing oxidative stress, maintaining fruit quality, and extending the storage time [27,45]. SOD is a naturally occurring scavenger of the superoxide free radical and can convert the superoxide radical into H_2_O_2_, which is ultimately converted into water by CAT and POD. Many postharvest treatments to preserve peach quality could induce greater activity of antioxidant enzymes [45,46,47]. As shown in Figure 8, the activities of SOD and CAT increased slightly in the earlier stage and then decreased in shelf life, which was similar to other studies [27,46]. The increase in enzyme activities might be induced by the stress response of cold temperature. At 9.75 d, after the third instance of an increase in temperature, the activity of the SOD enzyme in the VIC-10 group (44.24 U/g) was the highest, and the VIC-20 group (39.97 U/g) showed a significantly lower SOD activity than that of the constant-temperature and VIC-10 groups. In terms of shelf life, the SOD activity of the VIC-20 group was significantly lower than that of the VIC-10 and constant-temperature groups. At the same time, the CAT activity of the constant-temperature (18.02 U/g) and VIC-10 (17.61 U/g) groups was higher than that of the VIC-15 and VIC-20 groups. There were no significant differences in CAT enzyme activity between the VIC-20 (8.84 U/g) and VIC-15 (7.96 U/g) groups at the end of shelf life. The peach fruit of VIC-10 possessed higher POD activity at 2.5 d than the VIC-15 and VIC-20 groups. At 9.75 d, POD activity decreased significantly, and VIC-20 (1.92 U/g) and VIC-15 (1.89 U/g) peaches showed significantly lower activity than that of the constant-temperature group (2.34 U/g). When the peaches were transferred to shelf life, POD activity slightly increased. At the end of shelf life, both the constant-temperature and VIC-10 groups had significantly higher POD activity than the VIC-15 and VIC-20 groups. Storing peaches at an appropriately low temperature can decrease free radical content and inhibit the apoptosis of peaches, thereby delaying fruit ripening and senescence [27]. In summary, these three enzymes of VCC and VIC-10 peaches showed higher activities than the VIC-20 and VIC-15 groups from 9.75 d to the end of shelf life. The temperature rising to a higher value was unfavorable for maintaining enzyme activities. The SOD, POD, and CAT activities of peaches were highly active in peach fruit stored under lower temperatures than room temperature [27]. In the future, the activity of other antioxidant enzymes and their correlation with fruit quality should be considered to determine the depth of the impact of temperature abuse on the fruit stress response and antioxidant system.

### 3.8. Principal Component Analysis and Heatmaps of Peach Fruit in Different Virtual Cold Chains

**Figure 9:**
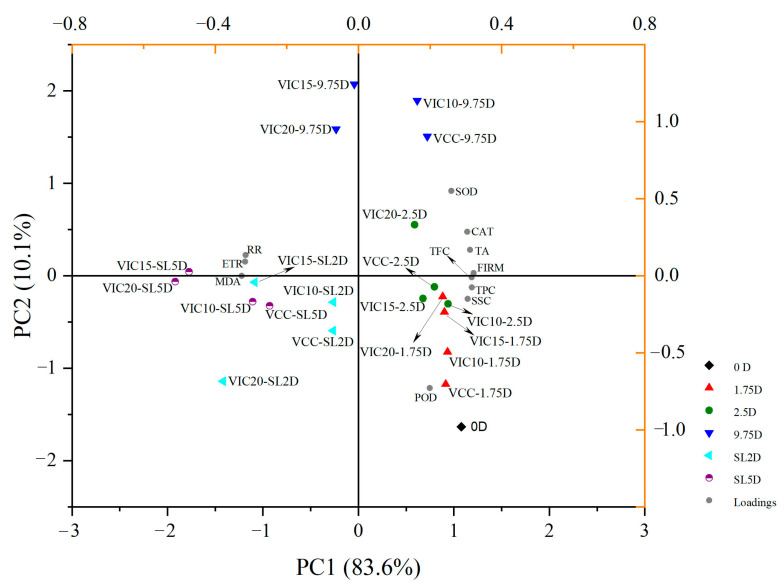
PCA biplot (score plot and loading plot) of peach fruits in different virtual cold chains. Dots represent peaches at different cold stages (0, 1.75, 2.5, and 9.75 days (D)). Peaches at day 2 and day 5 in shelf life are noted as SL2D and SL5D, respectively. The virtual incomplete cold chains with ambient temperature rising to 20, 15, and 10 °C are noted as VIC20, VIC15, and VIC10, respectively. VCC means a virtual complete cold chain with a constant temperature. RR, ETR, TPC, and TFC mean the respiratory rate, ethylene production rate, total phenol content, and total flavonoid content, respectively.

A principal component analysis was applied to describe all of the information contained in the dataset to detect the most important variables for the data structure. The main features of the PCA are the coordinates of the data in the new base (score plot) and the contribution to each component of the variators (loading plot) [48]. These can help to select a set of conditions with better quality performance. As shown in Figure 9, the first two principal components accounted for 93.7% of the total variance in peach quality along the virtual cold chains, in which PC1 explained 83.6%. Peaches stored at different virtual cold chains and times could be distinguished in the scores plot. Fresh peaches (0 D) and peaches stored for 1.75 and 2.5 days (except for the VIC20-2.5D) were separated in the bottom-right corner of the plot, meaning that the fruit qualities at these storage stages were similar to those of fresh fruits. The TPC, TFC, SSC, and POD were loaded in the same region, meaning that fresh peaches possess higher levels of these qualities. Furthermore, SOD, CAT, and TA were separated in the upper-right corner of the loading plot, mainly gathered with the peaches stored for 9.75 days, due to these qualities increased at this stage as shown in Figure 5B and Figure 8A,B. The respiratory rate, ethylene production rate, and MDA content were loaded on the negative axis of PC1, and the peaches in shelf life were clustered in the same side. This indicated that most of the fruit qualities decreased quickly when the peaches were stored under shelf conditions. In summary, peach quality was not very significantly affected when the transfer temperature increased to a lower value (10 °C) or suffered for a short time (2.5 d); however, as the storage time continued to 9.75 d, the temperature increase to 15 and 20 °C had significantly negative effects on peach quality, since several samples (VIC15-9.75D, VIC 20-9.75D, and VIC 15-SL5D) were separated in different axes with the constant-temperature and VIC-10 groups.

**Figure 10:**
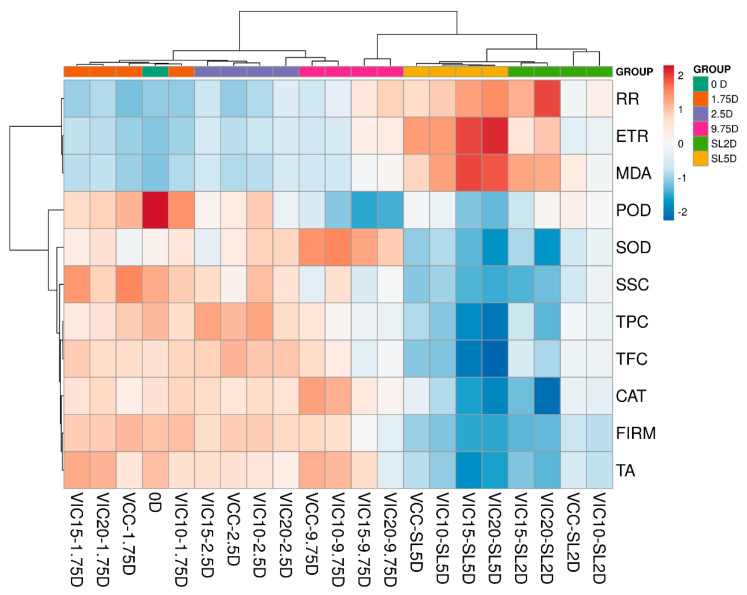
Heatmaps of peach qualities and different stages during storage in the virtual cold chains. Both rows and columns are clustered using correlation distance and average linkage. Dots represent peaches at different cold stages (0, 1.75, 2.5, and 9.75 days (D)). Peach of day 2 and day 5 in the shelf life are noted as SL2D and SL5D, respectively. The virtual incomplete cold chains with ambient temperature rising to 20, 15, and 10 °C are noted as VIC20, VIC15, and VIC10, respectively. VCC means a virtual complete cold chain with a constant temperature. RR, ETR, TPC, and TFC mean the respiratory rate, ethylene production rate, total phenol content, and total flavonoid content, respectively.

In order to visualize the evolution of peach qualities during the different virtual cold chains, a heatmap analysis was performed as shown in Figure 10. The highest POD activity was observed in the fruits at harvest (0D). The highest ethylene production rates and MDA contents were found in the peaches stored for 5 days in shelf life of the VIC-20 and VIC-15 groups, when the TPC and TFC were at their lowest levels. The cluster results of the heatmap were consistent with the PCA score plot. The peaches in shelf life were clustered into one group, and the peaches stored for 0, 1.75, and 2.5 days (except for VIC20-2.5D) were clustered as one group. For the variables, the ethylene production rate, respiratory rate, and MDA content were clustered together, which increased during storage compared to the other cluster composed of other qualities that decreased during the simulated cold chain.

## 4. Conclusions

Results from this study clearly showed that maintaining a constant temperature throughout a supply chain is paramount to maintain the good quality of peach fruit. Through different virtual cold chain experiments, increasing the ambient temperature of the loading and unloading stages to 15 and 20 °C three times would lead to the peach fruit core temperature increasing to a peak of 17.6 °C. The ethylene release rate and respiration rate were 21.03–28.16% and 12.36–15.89%, respectively, higher than the constant-temperature condition at the end of shelf life. Additionally, these temperature-rising conditions also accelerated peach fruit texture softening, with the lowest firmness of 3.84 kg/cm^2^ in the group where the temperature increased to 20 °C, and the lowering of acidity as well as polyphenol contents. The activities of antioxidant enzymes were significantly reduced in shelf life when peaches experienced increases in temperature to 15 °C and 20 °C along the supply chain, with the SOD, CAT, and POD activities being 6.03–15.52%, 31.74–38.53%, and 19.05–21.03% lower, respectively, than those of the constant-temperature group. A PCA and heatmap analysis also confirmed the results and suggested that the influence of an increase in temperature in transfer stages to a lower temperature (10 °C) on fruit quality was not particularly significant. Based on the results of this research, the temperature-rising threshold of several transfer stages of peach supply chains should be controlled not to exceed 10 °C to maintain a relatively stable circulation temperature environment, so as to ensure the qualities of peach fruit and ultimately reduce the loss of fruit.

## Data Availability

The data presented in this study are available on request from the corresponding author.
